# Phylogeographic dynamics of oropouche virus in the Colombian Amazon: Evolutionary insights in a climatic context

**DOI:** 10.1371/journal.pntd.0013810

**Published:** 2026-07-14

**Authors:** Laura S. Perez-Restrepo, Jaime Usuga, Alejandro Vasquez, Lina Yepes, Daniel Limonta, Cole Knuese, Isabel Moreno, Vanessa Vargas, Manuel Gonzalez-Ramirez, Michael G. Berg, Mary Rodgers, Gavin A. Cloherty, Juan P. Hernandez-Ortiz, Jorge E. Osorio

**Affiliations:** 1 Universidad Nacional de Colombia, sede Medellín, Colombia; 2 Abbott Pandemic Defense Coalition, Chicago, Illinois, United States of America; 3 School of Veterinary Medicine, University of Wisconsin, Madison, Wisconsin, United States of America; 4 Infectious Diseases Research, Abbott Diagnostics, Abbott Park, Illinois, United States of America; Faculty of Medicine - University of São Paulo, BRAZIL

## Abstract

**Background:**

In 2024, the largest RT-qPCR–confirmed outbreak of Oropouche virus (OROV) in 70 years occurred in the Brazilian Amazon, raising concerns about transmission and viral evolution in the neighboring Colombian Amazon Basin. This study characterizes the genetic diversity, epidemiology, and climatic context of OROV transmission during the 2024 outbreak in Leticia, Colombia.

**Methodology/principal findings:**

Through a large-scale febrile surveillance program, we detected 41 OROV-positive cases in Leticia in 2024. Molecular diagnostics, next-generation sequencing (NGS), and phylogenetic/phylogeographic analyses revealed the co-circulation of two distinct lineages. Fifteen sequences clustered within a lineage linked to previous outbreaks (PE/CO/EC-2008–2021), while 26 belonged to a reassortant lineage associated with the unprecedented 2024 outbreak (BR-2015–2024). This new reassortant lineage (BR-2015–2024) progressively displaced the traditional PE/CO/EC-2008–2021 lineage. Bayesian skyline analysis of 627 OROV genomes from Colombia and neighboring countries confirmed the decline of the historic lineage and the rapid expansion of the BR-2015–2024 lineage beginning in 2022, surpassing the former dominant strain by 2023. Time-series analyses further indicated that OROV outbreak peaks coincided with major ocean temperature transitions, specifically from La Niña to neutral conditions in early 2021 and from El Niño to neutral conditions in early 2024.

**Conclusions/significance:**

Our findings demonstrate the dynamic evolutionary landscape of OROV in the Colombian Amazon, with the emergence of a new reassortant lineage (BR-2015–2024) now driving transmission. The temporal correspondence between oceanic climate variability and outbreak timing suggests environmental factors may modulate OROV dynamics. These results emphasize the importance of integrated genomic surveillance and climate monitoring to strengthen outbreak underlying factors understanding and prediction and guide public health responses in the Amazon Basin.

## Author summary

OROV is an emerging arbovirus that causes febrile illness in tropical regions of South America. Although historically associated with the Amazon basin, recent outbreaks have confirmed its transmission beyond the Amazon. During the 2024 outbreak in Leticia, Colombia, we combined molecular diagnostics, genomic sequencing, and climate data to track viral spread and evolution. We identified the new reassortant OROV lineage (BR-2015–2024) that displaced the previously dominant strain, coinciding with shifts in ocean temperature patterns. These findings suggest that climate variability may influence OROV transmission cycles. By linking viral evolution with environmental factors, our study provides insights to improve outbreak forecasting and inform strategies to protect vulnerable populations in the Amazon.

## Introduction

OROV is a significant public health concern in Latin America [1]. In 2024, the largest RT-qPCR-confirmed OROV outbreak since its discovery in 1955 occurred, with over 16,000 confirmed and thousands of suspected cases [2]. For the first time, Guillain-Barré syndrome [3], vertical transmission [4,5], and fatalities [6] were linked to OROV, which typically causes febrile illness and, in some cases, encephalomyelitis [7].

A member of the *Orthobunyavirus* genus within the *Peribunyaviridae* family, OROV has a tripartite, single-stranded, negative-sense RNA genome composed of small (S), medium (M), and large (L) segments [8]. This genomic architecture not only dictates viral replication but also enables genetic reassortment, a mechanism that can drive rapid evolution and alter virulence [9,10]. The newly identified OROV BR-2015–2024 lineage, responsible for the unprecedented 2024 outbreak, highlights the virus’s capacity to expand beyond its historically recognized Amazonian range, with sustained transmission and human outbreaks reported in distinct extra-Amazonian biomes in Brazil and other parts of the Americas [6,11–16].

Humans primarily acquire OROV through bites from infected vectors, particularly the biting midge *Culicoides paraensis* [17,18], though additional arthropod species have been recently implicated [16,19]. While *C. paraensis* is the principal vector in urban settings, transmission in sylvatic cycles involves various insect species and vertebrate reservoirs, including primates, small mammals, and possibly birds [10,20]. Thriving in tropical environments, these vectors benefit from favorable conditions that enhance their proliferation and interactions with humans [20,21]. This intricate vector–host network complicates control efforts and highlights the importance of integrated epidemiological, entomological, and genomic surveillance strategies [22,23].

Since its initial identification in Trinidad and Tobago–just 11 km from mainland South America–in 1955 [24], OROV has demonstrated remarkable adaptability, causing outbreaks across Latin America [1,13]. While Brazil has reported most cases, Bolivia, Colombia, Cuba, Guyana, Peru, and the Dominican Republic experienced increasingly frequent outbreaks in 2024 [25]. Additionally, over 100 cases have been documented in travelers returning to the United States and Europe [2,26–31].

Recent studies suggest that global weather patterns influence OROV transmission dynamics [32–34]. Fluctuations in temperature, precipitation, and humidity, often associated with El Niño-Southern Oscillation (ENSO) shifts, can create conditions conducive to vector proliferation and viral spread [35]. While climatic factors are well-documented drivers of other vector-borne diseases [34,36,37], their role in OROV outbreaks remains underexplored.

This study presents a comprehensive phylogeographic analysis of OROV lineages in the southernmost Colombian Amazon Basin. By integrating newly generated genomic sequences with epidemiological and climatic data from January to November 2024, and supplementing these with retrospective insights [11,38], we examined the evolutionary dynamics of co-circulating OROV lineages. Our findings show that the newly identified lineage became predominant over a previously circulating lineage, marking the largest documented case series of two distinct OROV lineages within a confined region. Additionally, we assessed how climate variability influences transmission and delineated cross-border viral dissemination patterns. These findings provide crucial insights into the interplay between viral evolution, environmental context, and human activity, highlighting the urgent need for enhanced surveillance and targeted interventions to mitigate the public health impact of this neglected arboviral disease.

## Results

Between January and November 2024, acute-phase serum samples were collected from 689 patients in Leticia, a municipality in the Colombian Amazon. Patient ages ranged from 5 to 92 years, with a gender distribution of 52.6% male (363) and 47.4% female (326). Diagnostic testing using RT-qPCR and antigen-based rapid tests identified DENV in 3.0% (21/689) of cases and *Plasmodium spp*. in 23.65% (163/689). A single case of IV, AdV, and HRV was detected, while co-infections of DENV and malaria were observed in four cases (0.58%).

RT-qPCR detected OROV in 5.9% (41/689) of samples. All RT-qPCR–positive samples were subsequently subjected to viral isolation in *Aedes albopictus* C6/36 HT cell cultures. Following successful viral replication, viral RNA was extracted from infected cell culture supernatants and used for metagenomic next-generation sequencing on the Illumina platform using a 150 bp paired-end read protocol. Metagenomic sequencing was also attempted directly from RNA extracted from selected RT-qPCR–positive clinical samples; however, in several cases genome coverage and sequencing depth were insufficient for robust downstream analyses. Therefore, genomes used in this study were primarily obtained from viruses amplified through cell culture. Sequencing yielded high-quality libraries with uniform fragment size distribution and optimal sequencing concentrations. Near-complete genomes were obtained, with coverage exceeding 87% and an average depth of 48 × .

In addition to fever, OROV patients exhibited various non-specific symptoms. Headache was reported in 100% of cases, followed by bone ache and chills (87.8%), muscle pain (31.7%), abdominal pain (22%), diarrhea (19.5%), fatigue (17%), and, less frequently, odynophagia, jaundice, and rash (2.4%). Table 1 provides a comparative summary of demographics and clinical manifestations in patients infected with the two OROV lineages (PE/CO/EC-2008–2021 and BR-2015–2024). Chi-square analysis showed no significant differences in clinical presentation between the two lineages (S1 Table).

**Table 1:** Demographic characteristics and clinical manifestations of OROV-infected patients by lineage.

		PE/CO/EC 2008–2021 (n = 15)	BR 2015–2024 (n = 26)	Total (n = 41)
**Sex**	**Age**			
Male	>60y	0 (0.0%)	0 (0.0%)	15 (36.6%)
	36-60y	1 (6.7%)	2 (7.7%)	
	19-35y	4 (26.7%)	5 (19.2%)	
	<18y	1 (6.7%)	2 (7.7%)	
Female	>60y	1 (6.7%)	0 (0.0%)	26 (63.4%)
	36-60y	2 (13.3%)	7 (26.9%)	
	19-35y	3 (20%)	6 (23.1%)	
	<18y	3 (20%)	4 (15.4%)	
**Symptoms**				
Headache		15 (100%)	26 (100%)	41 (100%)
Diarrhea		4 (26.7%)	4 (15.4%)	8 (19.5%)
Skin Rash		0 (0.0%)	1 (3.8%)	1 (2.4%)
Muscle Pain		8 (53.3%)	5 (19.2%)	13 (31.7%)
Bone Pain		12 (80%)	24 (92.3%)	36 (87.8%)
Abdominal pain		1 (6.6%)	8 (30.8%)	9 (22%)
Chills		12 (80%)	24 (92.3%)	36 (87.8%)

**Fig 1:**
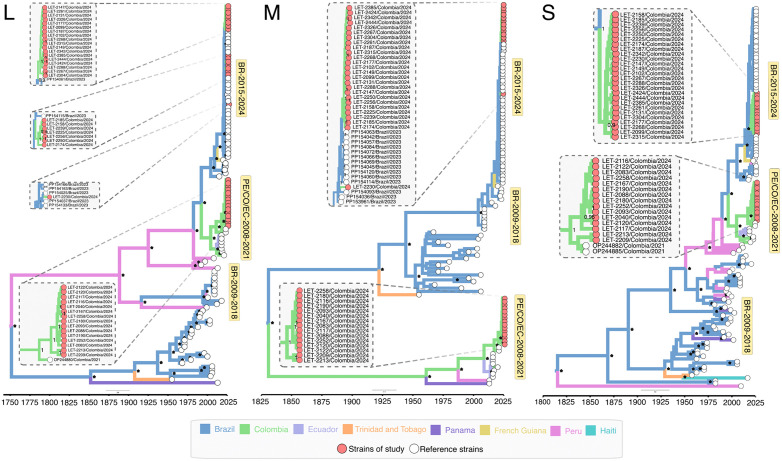
Phylogenetic analysis of the M, L, and S segments of OROV. Time-scaled Bayesian phylogeographic analysis of co-circulating OROV lineages in the Amazon Basin, Colombia, 2024. Bayesian phylogenetic trees for the large **(L)**, medium (M) and small (S) genomic segments were inferred using the Bayesian Markov chain Monte Carlo method (>100 million generations) in BEAST [39] and ModelFinder in IQ-TREE [40] with ultrafast bootstrapping (1,000 replicates). Red solid circles indicate viruses from this study, identified by the prefix “LET” for Leticia, Colombia. Branch colors represent the inferred geographic origins of descendant lineages. The best-fit model was selected based on Bayesian information criteria, and a strict molecular clock model was applied. Bayesian posterior probabilities (>0.9) are annotated with asterisks at relevant nodes. OROV sequences from this study were compared with reference sequences, with major clusters labeled as follows: BR-2015–2024 (Brazil), PE/CO/EC-2008–2021 (Peru, Colombia, and Ecuador), and BR-2009–2018 (Brazil). Scale bar represents nucleotide substitutions per site.

The phylogenetic analysis of the OROV L, M, and S segments sequenced from isolated viral samples in 2024 (Fig 1) revealed that the 41 sequences from our study were distributed into two distinct monophyletic clades. One clade comprising 26 sequences corresponded to the novel reassortant BR‑2015–2024 lineage, while the PE/CO/EC-2008–2021 clade contained the remaining 15 sequences.

**Fig 2:**
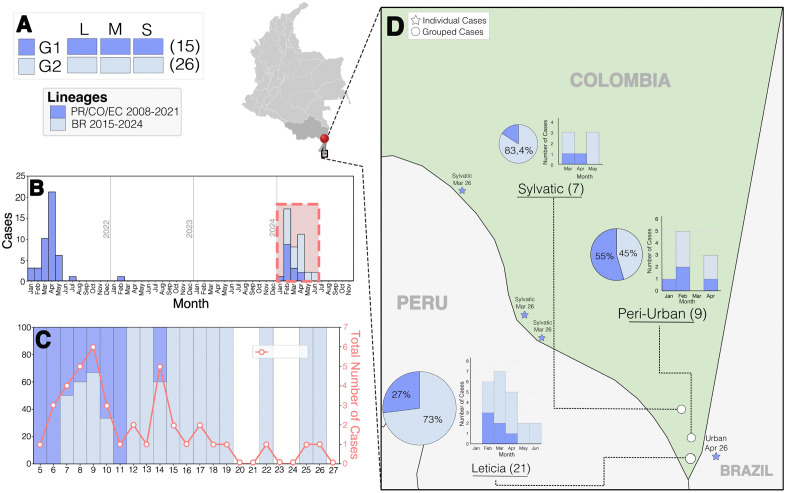
Oropouche outbreaks in Colombia during 2021–2024. **(A)** Genomic constellations of each gene segment for OROV samples collected in Leticia, Colombian Amazon Basin, in 2024. Colored boxes represent the two OROV lineages identified in this study. **(B)** Molecular identification of OROV lineages from January 2021 to December 2024. The red dashed line identifies the 2024 outbreak. **(C)** Temporal distribution of the two OROV lineages during a 27-week epidemiological period in 2024 in Leticia. **(D)** Geographical distribution of OROV cases in Leticia municipality and the Three Borders region (Colombia, Peru, and Brazil). Symbols indicate the residential locations of 41 patients infected with OROV in Leticia. Colored circles and diamonds represent patients infected with each OROV lineage. Maps were produced using QGIS Desktop 3.36.0 (QGIS Development Team, 2024). The base map for panel D was derived from Natural Earth public domain map data (https://www.naturalearthdata.com/downloads) and edited by the authors.

Fig 2 provides an overview of OROV outbreaks in Leticia from 2021 to 2024, illustrating the geographic and temporal distribution of two distinct viral lineages. Fig 2A shows that no new reassortment events were detected in the tri-segmented OROV genome, apart from the one previously reported by Naveca et al. [11], despite co-circulation in the Three Borders region. Dark blue (G1) bars represent samples where all genomic segments belonged to the PE/CO/EC-2008–2021 lineage, while light blue (G2) bars correspond to the newly identified BR-2015–2024 lineage. Fig 2B displays the monthly number of OROV cases in Leticia from January 2021 to November 2024. The red-dashed box highlights the 2024 outbreak, further detailed by epidemiological week in Fig 2C. In this panel, dark and light blue indicate the percentage of cases attributed to each lineage, while the red line represents the total weekly case count. The PE/CO/EC-2008–2021 lineage circulated from epidemiological weeks 5–14 in 2024, whereas the BR-2015–2024 lineage emerged in week 7 and dominated until the outbreak ended in week 27. Fig 2D maps OROV case distribution across urban, peri-urban, and sylvatic areas of Leticia. The BR-2015–2024 lineage was predominant in urban and sylvatic settings, while the PE/CO/EC-2008–2021 lineage was more common in peri-urban areas and remote indigenous communities, including Macedonia, Santa Sofía, and Yagua. Additionally, one case in Tabatinga, Brazil, near the Colombia-Brazil border, was linked to the PE/CO/EC-2008–2021 lineage, suggesting potential cross-border transmission.

Root-to-tip regression analyses revealed a positive correlation between genetic divergence and sampling time across all genomic segments (S1 Fig). The L and M segments exhibited stronger temporal signal (R² ≈ 0.73 and 0.72, respectively), whereas the S segment showed a moderate but consistent correlation (R² ≈ 0.46), supporting the use of molecular clock models in Bayesian analyses.

**Fig 3:**
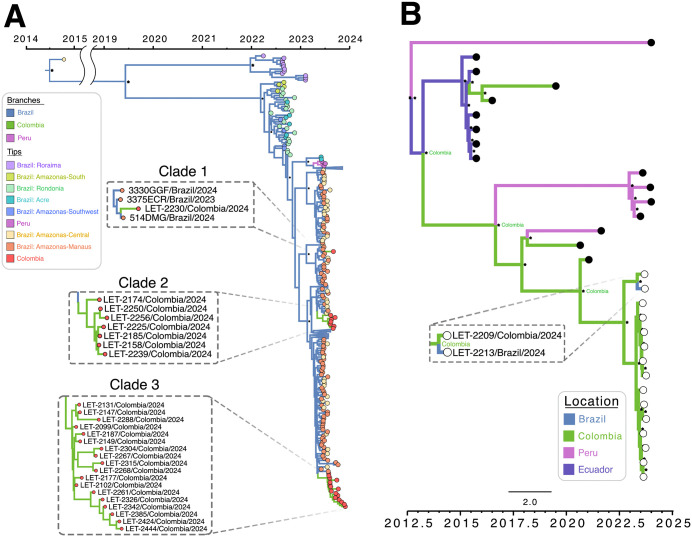
Bayesian phylogeographic analysis of two OROV lineages based on whole-genome concatenation. Bayesian time-scaled Markov chain Monte Carlo (MCMC) trees of the **(A)** BR-2015–2024 and **(B)** PE/CO/EC-2008–2021 OROV lineages were inferred from concatenated L, M, and S genomic segments using BEAST [42]. Branch colors represent the inferred ancestral locations estimated by the phylogeographic model, whereas tip colors indicate the sampling location of each sequence. In panel A, Brazilian reference sequences are further classified by subregion to distinguish central Amazonian localities (including Amazonas-Central and Manaus) from western Amazonian localities closer to the Colombia–Brazil border (including Amazonas-Southwest and Acre). Red circles indicate strains generated in this study, whereas the remaining tips correspond to reference strains. Phylogenetic reconstruction was performed using a Bayesian MCMC approach (>100 million generations), and the best-fit substitution model was selected using ModelFinder in IQ-TREE [43] according to the Bayesian information criterion. A strict molecular clock model was applied. Bayesian posterior probabilities >0.9 are indicated by asterisks at supported nodes. Because OROV is a segmented virus, this concatenated analysis is presented as a complementary genome-wide view to the segment-specific phylogenies shown in Fig 1. Scale bar represents time in years.

The Bayesian phylogeographic analysis of a full concatenated genome alignment of the novel reassorted OROV BR-2015–2024 lineage (Fig 3A), revealed multiple independent introductions into Colombia. The 2024 sequences clustered into three main clades (Clade 1, Clade 2, and Clade 3), which traced their ancestry to Brazil. After incorporating finer geographic annotation of Brazilian reference sequences, these Colombian clades were found to cluster more closely with strains sampled from central Amazonas, including Manaus and Amazonas-Central, than Amazonas-Southwest and Acre. This pattern suggests that viral dissemination into Leticia may have been associated more strongly with human mobility and fluvial connectivity along the Amazon River corridor [41] than with short-range spread, although this interpretation should be viewed cautiously given the uneven regional availability of Brazilian genomes. Clade 1 comprises a single strain (LET-2230). Clade 2 was introduced in April of 2023 and subsequently disseminated into seven strains. Clade 3, the largest viral group with 18 strains, includes both the earliest strains collected in February of 2024 as previously reported by Usuga et al. in 2024 [38], and the latest strains collected in late June of 2024 and reported in this study. This phylogeographic tree suggests that Clade 3 was the first to be introduced into Colombia, with an estimated entry date in March of 2023, approximately 11 months before its detection in Leticia. To further quantify the temporal structure of these introduction events, we analyzed the posterior distributions of tMRCA for each clade (S2 Fig). The violin plots revealed a temporal gradient among clades, with Clade 3 showing the earliest tMRCA estimates, followed by Clade 2 and subsequently Clade 1. The distributions displayed partial separation with limited overlap in their 95% HPD intervals, supporting the occurrence of multiple independent introductions of the BR-2015–2024 lineage into Colombia.

A phylogeographic tree based on concatenated genes from the PE/CO/EC-2008–2021 lineage (Fig 3B) was constructed using genomic sequences from Peru, Ecuador, Colombia, and Brazil. This analysis revealed that Colombian sequences, including those from this study, formed two main clades originating from Ecuador. The upper clade was detected in northern Colombia in 2016 and 2020, while the lower clade was found in Leticia and later reemerged in Colombia in 2020 and 2021 [43] and during the 2024 outbreak [38], which included 15 sequences from this study.

Further analysis showed that the strains from this study clustered into two subclades (Fig 3B): one containing 13 strains and the other containing two, including one from Tabatinga, Brazil. Phylogeographic reconstruction suggests that this strain entered Brazil from Colombia, highlighting frequent lineage exchange across the Brazil-Colombia border. Notably, this is the first report of this lineage in Brazilian territory.

**Fig 4:**
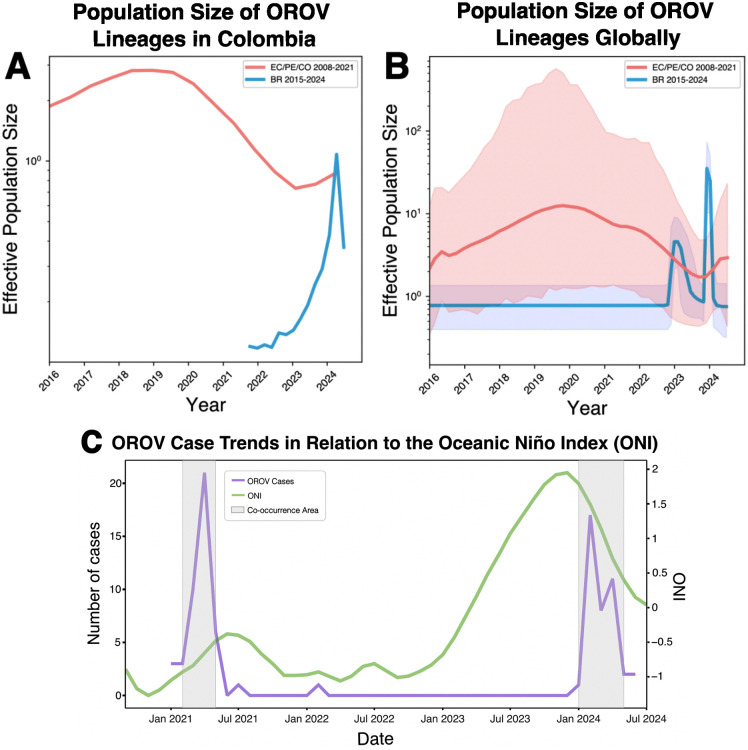
Impact of Population Dynamics and Global Climate Patterns on OROV outbreaks in the Colombian Amazon Basin. The demographic history of the two circulating OROV lineages, PE/CO/EC-2008–2021 and BR-2015–2024, are displayed in **(A)** Colombia and (B) across Latin America and other places. Both plots illustrate the estimated effective number of OROV infections (y-axis) over time (x-axis), inferred using the coalescent-based Bayesian Skygrid (BSKG) model. **(C)** OROV cases in Leticia, Colombia, alongside ONI values from 2021 to 2024. The blue line represents the number of OROV cases, while the red line shows ONI values. Gray-shaded periods indicate transitions from El Niño/La Niña conditions to neutral ENSO conditions, highlighting a notable correlation between OROV outbreaks and the climatic shifts.

To investigate OROV’s population dynamics, we estimated the effective population size (EPS), a measure of genetic drift, over time using Bayesian skyline analysis. Two datasets were analyzed: one with sequences exclusively from Colombia (Fig 4A) and another including sequences from Latin America and beyond (Fig 4B). The PE/CO/EC-2008–2021 lineage exhibited a gradual EPS decline from 2016 to 2021 in both datasets, followed by a sharp decrease at the end of the study period, indicating reduced genetic diversity and prevalence. In contrast, the BR-2015–2024 lineage showed a rapid EPS increase beginning in 2022, surpassing the earlier lineage by 2023. This expansion was evident in Colombia and globally, though the global dataset exhibited broader credible intervals and greater fluctuations in 2022–2024, likely reflecting sampling heterogeneity and higher genetic diversity across regions (Fig 4A-B).

Fig 4C shows a time series of OROV cases in Leticia (left y-axis) and ONI, right y-axis from January 2021 to July 2024, with gray shaded regions denoting correlation in trends of OROV cases and ONI in early 2021 and early 2024. During early 2021, OROV cases peaked sharply (exceeding 15 cases) as the ONI shifted from La Niña (negative values) to neutral conditions. After a prolonged period of low viral detection, OROV cases rose again in early 2024, coinciding with the ONI’s transition from El Niño (positive values) back to neutral conditions. These findings suggest that abrupt changes in ENSO phases, marked by fluctuations in precipitation and temperature, may create favorable conditions for vector proliferation and subsequent OROV transmission.

Finally, a Mann-Whitney U test revealed a highly significant difference in the 3-month climatic trajectories between outbreak (n = 10) and non-outbreak months (n = 29) (U = 31.0, p = 0.0003). Specifically, outbreak months were strongly associated with a rapid, negative shift in the 3-month rolling distance from the neutral zone (median 3-month change = -0.36), indicating that the climate was abruptly transitioning away from El Niño or La Niña extremes and converging toward neutral conditions. In contrast, non-outbreak months exhibited a slight divergence away from the neutral zone (median 3-month change = 0.11), representing periods of sustained climatic stability in either extreme phase.

## Discussion

The co-circulation of two distinct OROV lineages during the 2024 outbreak, PE/CO/EC-2008–2021 and BR-2015–2024, underscores the complexity of the outbreak. The early predominance of PE/CO/EC-2008–2021, followed by the sustained predominance of the novel BR-2015–2024 strain after March, suggests lineage replacement during the course of the outbreak. Additionally, the observed patterns are consistent with a potential link between OROV reemergence and climatic variability, highlighting the role of environmental factors in outbreak dynamics.

The demographic and clinical profile of the 41 confirmed OROV cases suggests a possible susceptibility among women and among individuals in the 11–18 and 19–25 age groups, potentially influenced by occupational, behavioral, or environmental exposure to vectors. The predominant symptoms—headache, bone ache, and chills—closely resemble those of other endemic arboviral infections like dengue [44], underscoring the need for differential laboratory diagnosis. Although recent studies have reported vertical transmission and fatalities linked to OROV [4–6], no severe cases or pregnancies were identified in this study. The absence of co-infections further supports OROV as the primary causative agent, reinforcing the importance of continued surveillance to assess long-term health impacts, such as Guillain-Barré syndrome [3].

Phylogenetic analysis of OROV strains in the Colombian Amazon reveals the presence of both BR-2015–2024 and PE/CO/EC-2008–2021 lineages, highlighting the genetic diversity and adaptability of OROV Orthobunyaviruses in the region. To further elucidate their evolutionary origins, we employed a comparative framework incorporating three representative lineages (BR-2008–2019, PE/CO/EC-2008–2021, and BR-2015–2024) following the classification by Naveca et al. [11]. This approach enabled a segment-specific analysis of the L, M, and S genomic components, revealing key insights into the emergence of the BR-2015–2024 lineage.

As shown in Fig 3A, multiple independent introductions of the BR-2015–2024 lineage occurred in Leticia, with some leading to sustained circulation and evolution. The L and S segments of this lineage share a monophyletic relationship with PE/CO/EC-2008–2021, whereas the M segment demonstrates a paraphyletic relationship, suggesting reassortment. Our phylogeographic analysis indicates that the 2024 reemergence of PE/CO/EC-2008–2021 in Leticia resulted from local persistence rather than external introductions, with a close phylogenetic link to strains reported in 2021 (Fig 3B). Additionally, multiple introduction events from Colombia into Peru were detected, with one originating in 2017 and another in 2016, later reemerging during the 2024 outbreak [45]. Importantly, the analysis of posterior tMRCA distributions provides additional support for these independent introduction events. By incorporating the full posterior uncertainty, rather than relying solely on point estimates from the maximum clade credibility tree, we demonstrate a consistent temporal separation among clades. This strengthens the inference that these viral groups were introduced into Colombia at different time points, rather than arising from local diversification following a single introduction.

Bayesian phylogenetic analysis supports the reassortment event identified by Naveca et al. [11], who used a maximum likelihood estimation (MLE) approach, whereas our study employed a Bayesian time-scaled Markov chain Monte Carlo method. Both analyses converge on the conclusion that the BR-2015–2024 lineage likely arose through reassortment involving the M segment from the BR-2008–2019 lineage. This highlights the dynamic evolutionary processes shaping OROV diversity and underscores the importance of segment-specific phylogenies in viral evolution studies.

Our phylogeographic analysis (Fig 3A) further reveals that BR-2015–2024 gave rise to three distinct groups—Clade 1, Clade 2, and Clade 3—following its multiple introductions into Colombia. Notably, Clade 3 exhibits the broadest dissemination, suggesting enhanced infectivity and adaptability, making it a likely driver of BR-2015–2024 lineage expansion, consistent with previous reports [11]. Notably, the Colombian BR-2015–2024 clades were more closely related to sequences from central Amazonas/Manaus than to sequences from other Brazilian areas (Acre and southwestern Amazonas). Although limited by the geographic heterogeneity of available genomes, this pattern is more consistent with long-distance dissemination through river-connected population corridors than with gradual spread through adjacent forest-border areas. Additionally, a PE/CO/EC-2008–2021 strain (LET-2213/Brazil/2024) was introduced into Brazil from Colombia, demonstrating cross-border viral exchanges between Leticia, Colombia, and Tabatinga, Brazil—likely facilitated by human mobility and trade. This marks the first documented occurrence of this OROV strain in Brazil, warranting further investigation into its local transmission potential and public health implications.

Previous research [38] identified mutations in the RNA-dependent RNA polymerase and glycoproteins of BR-2015–2024, key components for replication efficiency and immune evasion. These mutations may have contributed to the lineage’s increased transmissibility, as evidenced by its predominance in the last 15 weeks of the outbreak. This raises critical questions about cross-protective immunity between OROV lineages, particularly given the limited cross-protection observed in other reassortant strains [1,46]. The co-circulation of multiple OROV lineages in the same region also heightens the risk of reassortment [47], potentially leading to the emergence of more virulent strains with enhanced vector competence. Notably, recent reports describe the co-circulation of these two lineages in the Peruvian Amazon, though occurring in separate cities over 1000 km apart [15].

Population dynamics analysis reveals contrasting evolutionary trajectories for the two lineages. The PE/CO/EC-2008–2021 lineage exhibited a gradual decline in genetic diversity and prevalence, possibly due to herd immunity [48] or competition with emerging variants [1,45,49,50]. In contrast, BR-2015–2024 displayed a sharp increase in EPS starting in 2022, surpassing PE/CO/EC-2008–2021 by 2023. This trend, observed both in Colombia and across Latin America (Fig 4A-B), suggests that enhanced fitness, transmission potential, or immune evasion mechanisms favor BR-2015–2024’s persistence and expansion [51–53]. The substantial uncertainty in the Colombian dataset (Fig 4A) highlights the need for expanded genomic surveillance to better characterize OROV diversity and transmission dynamics.

ENSO-related climatic variability significantly influences vector breeding sites [36,54], likely shaping OROV transmission patterns, particularly in riverine environments. Increased rainfall during ENSO transitions can lead to flooding and stagnant water formation, fostering vector proliferation [37,55]. Conversely, droughts can create isolated water pools that sustain high vector densities. These hydrological shifts may amplify OROV transmission by prolonging viral circulation in human populations [33]. The temporal correlation between ENSO events and OROV outbreaks (Fig 4C) underscores the need for further research to establish causal links and integrate climate factors into predictive models for outbreak mitigation.

The highly significant association (p = 0.0003) between 3-month climate transitions toward neutral ONI conditions and active OROV transmission, supporting the visual time-series observations. A 3-month evaluation window aligns with the biological plausibility of vector ecology, providing the necessary lag time for Culicoides midges to breed following abrupt hydrological shifts. However, we acknowledge the limitations of evaluating climatic drivers based on two episodic outbreaks. While this non-parametric approach supports a strong statistical association with ENSO transitions, a dataset capturing only two major peaks is underpowered for robust multivariable time-series regression. Consequently, this analysis cannot account for autocorrelated, confounding covariates such as daily micro-variations in precipitation or relative humidity. These findings are therefore observational and hypothesis-generating, underscoring the need for continuous eco-epidemiological surveillance in the Amazon Basin [56].

Consistent with previous findings [57], our study shows peak OROV transmission during the Amazonian rainy season, reinforcing the role of environmental factors—such as deforestation and climate change—in facilitating viral spread. Moreira et al. [58] emphasized that deforestation in Amazonian frontier regions contributes to OROV transmission by disrupting ecosystems and increasing human-vector interactions. Our data support this, as OROV cases in Leticia were detected across sylvatic, peri-urban, and urban settings (Fig 2D), where human encroachment into altered environments may elevate infection risks.

Although entomological surveillance was beyond the scope of this study, vector context is important for interpreting OROV transmission in Leticia. *Culicoides paraensis* is the main recognized urban vector, but other arthropods have also been investigated (*Culex spp.*). However, the specific vectors involved in local transmission remain poorly characterized, highlighting the need for targeted entomological studies in the Colombian Amazon [59].

The interplay between viral evolution and environmental factors presents significant challenges for OROV control in the Americas [10,60,61]. The rapid rise of BR-2015–2024, with its apparent ecological advantages, suggests the potential for further expansion and reassortment, potentially leading to increasingly transmissible or virulent strains. Continued genomic surveillance, combined with robust epidemiological and ecological monitoring, is essential for understanding the impact of climate variability on OROV transmission and mitigating future outbreak risks.

Our study documents the displacement of the reemerging PE/CO/EC-2008–2021 lineage by multiple independent introductions and the sustained transmission of the novel BR-2015–2024 lineage in the Colombian Amazon Basin in 2024. These findings underscore the complex evolutionary dynamics driving OROV diversity and the potential for genetic reassortment events to shape outbreak patterns. Additionally, we provide strong evidence linking ENSO-related climate variability—a global weather phenomenon—to OROV transmission rates in the Colombian Amazon. The observed correlation between shifts in the ONI and outbreak peaks suggests that environmental factors, such as fluctuations in precipitation and temperature, significantly influence vector breeding and disease propagation. Overall, this study highlights the critical need for a multidisciplinary approach that integrates genomic surveillance with climatic and ecological monitoring. Strengthening these efforts will be essential for predicting, detecting, and mitigating future OROV outbreaks in vulnerable regions across the Americas.

## Methods

### Ethics statement

Written informed consent was obtained from all adult participants before epidemiological data were collected through a survey. For participants under 18 years of age, written informed consent was obtained from a parent or legal guardian, and assent was obtained from the minors. The study was approved by the Ethics Committee of the Corporación para Investigaciones Biológicas (CIB 10102022).

### Study design and participant recruitment

During a year-long surveillance of acute febrile illness (AFI) in Leticia, Colombian Amazon region, in 2024, OROV disease was documented. While we performed an initial report of eight OROV cases in January and February [38], the present work combined data collected from January through November 2024. No new cases of OROV were identified after June. Participants ranged from 6 to 92 years of age.

The study included patients presenting AFI, defined as a body temperature exceeding 38°C at the time of medical consultation, symptom onset within the previous seven days, and no identified source of infection. Non-specific clinical symptoms, including headache, rash, and muscle and joint pain, were collected. These patients were assessed at the emergency department of San Rafael de Leticia Hospital in Leticia.

Upon recruitment, blood samples were collected for rapid diagnostic tests targeting dengue virus or DENV (SD Bioline Dengue Duo, Abbott), malaria (SD Bioline Malaria Ag P.f/Pan, Abbott), and nasopharyngeal swabs were collected for severe acute respiratory syndrome coronavirus 2 or SARS-CoV-2 (Panbio COVID-19 Ag Rapid Test Device), and influenza virus or IV (Bioline Influenza Antigen). Serum, plasma, and swab samples were stored at -80°C and subsequently shipped by air to the One Health Genomic Lab at the Universidad Nacional de Colombia in Medellín for advanced molecular analyses.

At the One Health laboratory, RT-qPCR and qPCR assays were performed to detect a number of human pathogens, including *Plasmodium spp*, DENV, Zika virus (ZIKV), chikungunya virus (CHIKV), IV, adenovirus (AdV), SARS-CoV-2, and OROV. These molecular assays followed previously published protocols from other laboratories [62–66] and were optimized by our laboratory.

### Study area and local climatology

The study was conducted in Leticia, a municipality located in the southernmost region of the Colombian Amazon Basin. Because it is situated in a deep equatorial zone, the region features a tropical rainforest climate that does not experience traditional temperate seasons such as spring or summer. Instead, the local climatology is characterized by precipitation-driven cycles, typically divided into rainy and relatively dry seasons, with consistently high temperatures and relative humidity year-round. These baseline hydrological patterns are heavily modulated by the ENSO. Abrupt transitions between ENSO phases, as measured by shifts in the Oceanic Niño Index (ONI), disrupt normal weather patterns, causing severe fluctuations in local rainfall and temperature. These sudden hydrological shifts—ranging from abnormal flooding and stagnant water formation during periods of excess rainfall to the creation of isolated water pools during drought conditions—foster optimal breeding habitats for Culicoides midges and other potential arthropod vectors. Consequently, these transitional climatic disruptions create highly favorable environments for vector proliferation, increasing the probability of human-vector interactions and subsequent Oropouche virus (OROV) transmission [36,67,68].

### Virus isolation and RT-qPCR confirmation

Following confirmation of OROV infection by RT-qPCR in blood samples, virus isolation was performed with the *Aedes albopictus* C6/36 cell line. Patient serum (500 µL) was diluted 1:99 in Leibovitz’s medium (L-15) supplemented with 5% fetal bovine serum (FBS). The diluted serum was added to a 25 cm² flask containing an ~ 80% confluent monolayer of C6/36 cells. To facilitate viral adsorption, the cells were incubated at 28°C for one hour. Next, the medium was replaced with L-15 medium containing 5% FBS, 1% penicillin-streptomycin, and 0.025% amphotericin. The cultures were incubated at 28°C for up to 10 days or until ~70% cytopathic effect (CPE) was observed. After the isolation protocol, the supernatant was collected, and OROV replication was confirmed by RT-qPCR, as described previously [43]. The molecular analyses were conducted by the 7500 Fast Real-Time PCR System (Applied Biosystems).

### Metagenomic Next-Generation Sequencing (mNGS)

For downstream analysis, RNA was extracted from infected C6/36 HT cell cultures for metagenomic next-generation sequencing (mNGS). Prior to nucleic acid extraction, samples were treated with Benzonase nuclease (Sigma-Aldrich) to degrade unprotected host nucleic acids originating from the mosquito cell line, thereby enriching viral nucleic acids protected within viral particles.

The sequencing workflow utilized a MiSeq platform and the Nextera XT kit (Illumina, California, USA), following the protocol reported by Berg et al. [42]. Total RNA obtained after nuclease treatment was directly used as input for library preparation, and no additional rRNA depletion step was performed.

Library preparation included DNA quantification with a Qubit 4.0 fluorometer (Thermo Fisher Scientific) and fragment size assessment using the Agilent High Sensitivity DNA kit on a 4150 TapeStation (Agilent Technologies), according to the manufacturer’s instructions. Libraries were pooled at a specific concentration and loaded onto the MiSeq platform for sequencing.

### Data curation, phylogenetic and phylogeographic analysis

Sequence alignment and consensus sequence generation were performed by mapping reads to segment-specific OROV reference genomes using BWA v0.7.17 and Samtools v1.15 [69,70]. The GenBank sequences AF484424, AF441119, and AY237111 were used as reference genomes for the S, M, and L segments, respectively. Available sequences of OROV as of June 2024 were obtained from the NextStrain repository [71]. These sequences, along with the identified strains in this study, were aligned using MAFFT v7.453 [72]. Phylogenetic analysis was performed using the maximum likelihood (ML) approach in IQ-TREE2 [40]. To assess the presence of temporal signal prior to Bayesian phylogenetic inference, root-to-tip regression analyses were performed using TempEst for each genomic segment (S, M, and L). Maximum likelihood trees were used as input, and the correlation between genetic divergence and sampling time was evaluated. To determine the temporal emergence and evolutionary rate of OROV, a time-scaled phylogeny was constructed with BEAST 1.10.5, utilizing the BEAGLE 3 library to enhance computational efficiency [43]. Molecular clock parameters were applied based on previously established frameworks. Posterior tMRCA distributions for each clade within the BR-2015–2024 lineage were extracted from the BEAST posterior tree set (.trees file) using node heights and visualized with violin plots and 95% HPD intervals.

To evaluate the impact of ENSO on the 2024 OROV outbreak in Colombia, we analyzed sea surface temperature (SST) anomaly data from the Climate Prediction Center of the National Oceanic and Atmospheric Administration (CPC-NOAA, Maryland, US). The three-month running mean of the Oceanic Niño Index (ONI) for the Niño 3.4 region (120°W-170°W, 5°N-5°S) was accessed via the CPC-NOAA website [71] on December 9, 2024. This index, derived from SST anomalies in the Niño 3.4 region, served as a proxy for ENSO conditions. ONI values were classified as El Niño (+0.5 or higher), La Niña (-0.5 or lower), or neutral (between -0.5 and +0.5), enabling the identification of ENSO phases during the study period. We then correlated ONI values with OROV cases detected by our group in Leticia in 2021 [43] and 2024 [38], along with the cases reported in this study.

### Climatic data and statistical analysis

To evaluate the relationship between ENSO conditions and OROV emergence, we conducted a retrospective, non-parametric analysis. We categorized the surveillance period into “Outbreak Months” (months with ≥1 confirmed OROV case) and “Non-Outbreak Months” (zero confirmed cases). To capture the biological lag of vector proliferation during climatic shifts, we calculated the 3-month rolling change in the ONI’s absolute distance from the established neutral zone (-0.5 to +0.5). We utilized the Mann-Whitney U test [73] to compare this 3-month climatic trajectory between outbreak and non-outbreak periods, testing the hypothesis that dynamic transitions toward neutral ENSO conditions favor active viral transmission.

A Chi-square test was conducted to compare symptoms among patients infected with the PE/CO/EC-2008–2021 and BR-2015–2024 lineages. This statistical analysis tests for potential differences in clinical manifestations between the two lineages, providing insights into differences in symptoms and possible lineage-specific clinical phenotypes.

## Supporting information

S1 Fig Root-to-tip regression analyses for OROV genomic segments S, M, and L. Analyses were performed using TempEst v1.5.3 on maximum likelihood trees. Each point represents a sequence, with genetic divergence plotted against sampling time. The regression line and corresponding R² value indicate the strength of the temporal signal for each segment.
(DOCX)

S2 Fig Posterior distribution of tMRCA for each introduced clade within the BR-2015–2024 lineage. Violin plots show the posterior distributions of the time to the most recent common ancestor (tMRCA) for each identified clade (Clades 1–3) within the BR-2015–2024 lineage in Colombia. Distributions were obtained from the BEAST posterior tree set by extracting node heights corresponding to the most recent common ancestor of each clade across the posterior sample. Violin width represents the density of tMRCA estimates, with wider regions indicating higher posterior probability. Central points indicate the median estimate, and boxes represent the 95% highest posterior density (HPD) intervals. The temporal distribution of tMRCA estimates suggests a gradient of introduction events, with Clade 3 representing the earliest introduction, followed by Clade 2 and then Clade 1. The partial separation and limited overlap among distributions support multiple independent introductions of the BR-2015–2024 lineage into Colombia.
(DOCX)

S1 Dataset Supplementary dataset used in this study. Excel workbook containing three sheets: (i) “Data per Case”, with de-identified epidemiological, demographic, clinical, and diagnostic data for confirmed OROV cases; (ii) “ONI”, with Oceanic Niño Index (ONI) values used for climatic analyses; and (iii) “Bioinfo-Data”, with sequencing and assembly metrics for OROV genomic segments, including genome size, sequencing depth, coverage, mapped reads, total reads, and the accession number corresponding to each genomic segment and sample. Raw sequencing reads generated in this study have been deposited in the NCBI Sequence Read Archive (SRA) under accession numbers **SRR37531510–SRR37535986**. Consensus sequences generated in this study are associated with GenBank accession numbers **PQ146147–PQ146181**, **PQ146445–PQ146479**, and **PZ149237–PZ149271**.
(XLSX)

S1 File Multiple sequence alignments used for phylogenetic analyses. Compressed (.zip) file containing nucleotide alignments for OROV genomic segments (S, M, and L) and used in maximum likelihood and Bayesian phylogenetic inference. Alignments are provided in FASTA format and include both newly generated sequences from this study and reference sequences retrieved from GenBank.
(ZIP)

S1 Table Chi-square comparison of clinical manifestations between OROV lineages. Clinical manifestations were compared between patients infected with the PE/CO/EC-2008–2021 lineage and those infected with the BR-2015–2024 lineage using chi-square analysis. No statistically significant differences were observed between lineages.
(DOCX)
