# Supplementary material for: Phylogeographic dynamics of oropouche virus in the Colombian Amazon: Evolutionary insights in a climatic context
Source: PLoS Negl Trop Dis. 2026 Jul 14;20(7):e0013810. doi: 10.1371/journal.pntd.0013810 (PMC13387556; doi:10.1371/journal.pntd.0013810)
Supplement: S1 Fig — Analyses were performed using TempEst v1.5.3 on maximum likelihood trees. Each point represents a sequence, with genetic divergence plotted against sampling time. The regression line and corresponding R² value indicate the strength of the temporal signal for each segment. (DOCX) [file pntd.0013810.s001.docx]

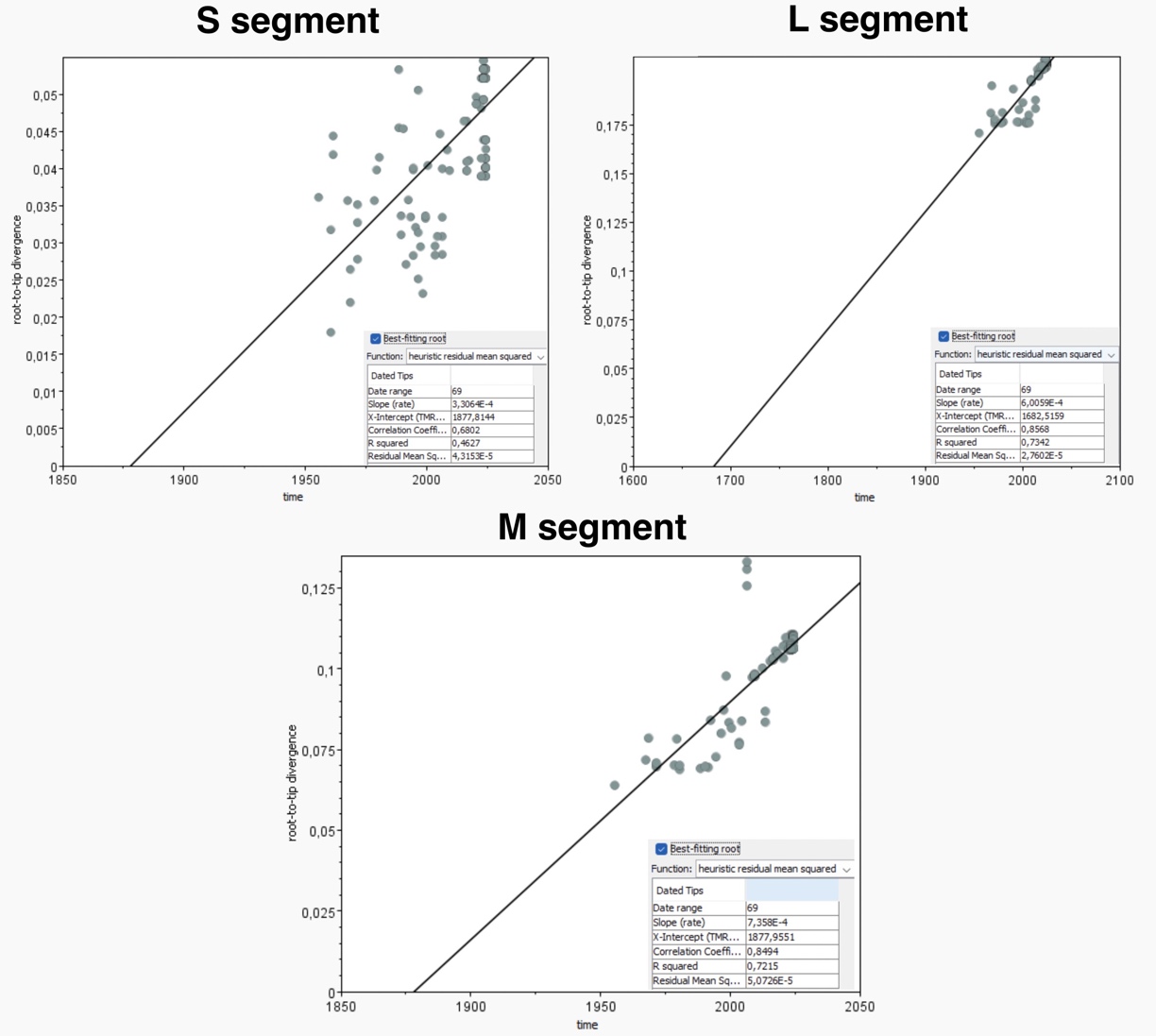


**S1 Fig. Root-to-tip regression analyses for OROV genomic segments S, M, and L.** Analyses were performed using TempEst v.1.5.3 on maximum likelihood trees. Each point represents a sequence, with genetic divergence plotted against sampling time. The regression line and corresponding R² values indicate the strength of the temporal signal for each segment.
