# Supplementary material for: Phylogeographic dynamics of oropouche virus in the Colombian Amazon: Evolutionary insights in a climatic context
Source: PLoS Negl Trop Dis. 2026 Jul 14;20(7):e0013810. doi: 10.1371/journal.pntd.0013810 (PMC13387556; doi:10.1371/journal.pntd.0013810)
Supplement: S2 Fig — Violin plots show the posterior distributions of the time to the most recent common ancestor (tMRCA) for each identified clade (Clades 1–3) within the BR-2015–2024 lineage in Colombia. Distributions were obtained from the BEAST posterior tree set by extracting node heights corresponding to the most recent common ancestor of each clade across the posterior sample. Violin width represents the density of tMRCA estimates, with wider regions indicating higher posterior probability. Central points indicate the median estimate, and boxes represent the 95% highest posterior density (HPD) intervals. The temporal distribution of tMRCA estimates suggests a gradient of introduction events, with Clade 3 representing the earliest introduction, followed by Clade 2 and then Clade 1. The partial separation and limited overlap among distributions support multiple independent introductions of the BR-2015–2024 lineage into Colombia. (DOCX) [file pntd.0013810.s002.docx]

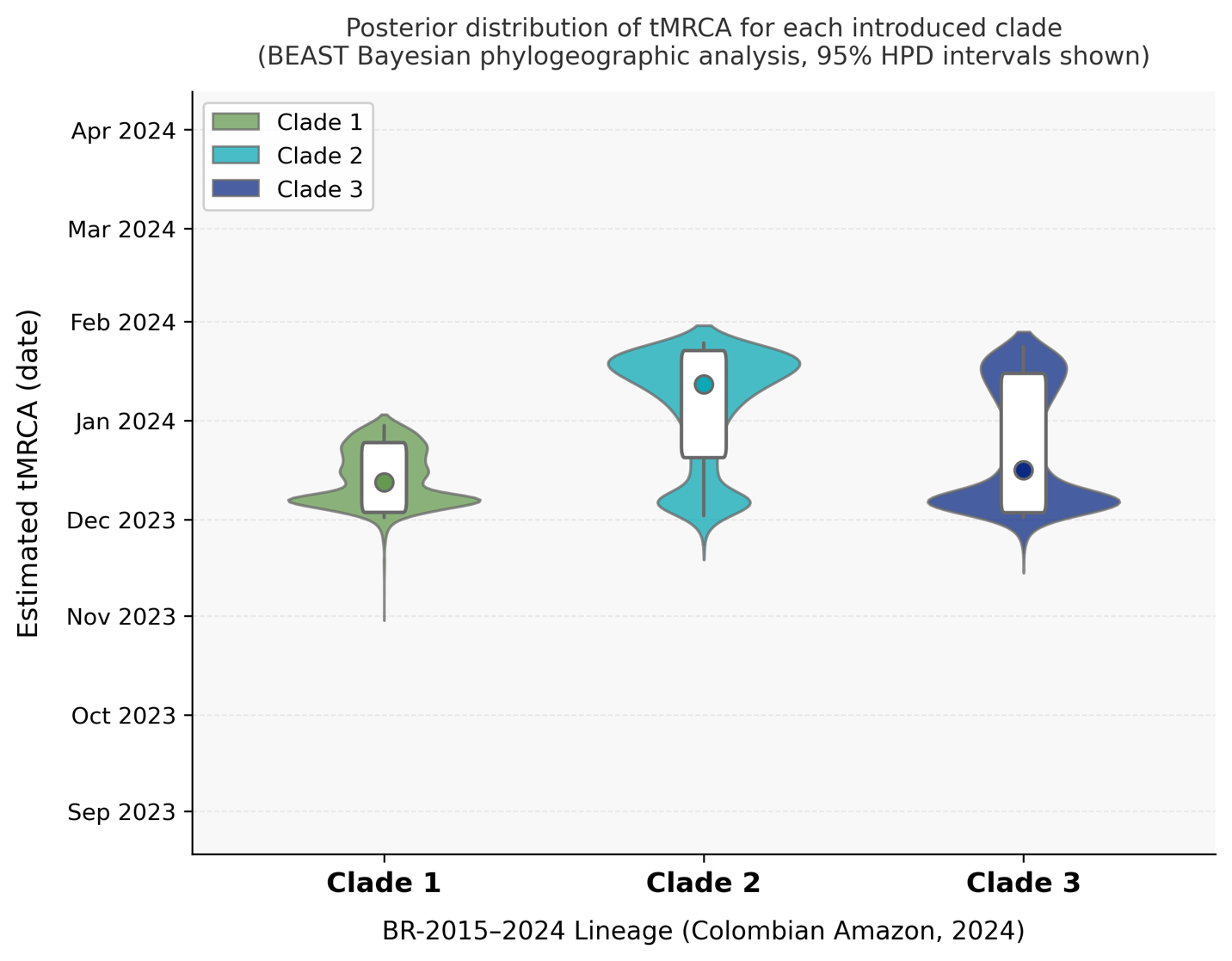


**S2 Fig. Posterior distribution of tMRCA for each introduced clade within the BR-2015–2024 lineage** Violin plots showing the posterior distributions of the time to the most recent common ancestor (tMRCA) for each identified clade (Clade 1–3) within the BR-2015–2024 lineage in Colombia. Distributions were obtained from the BEAST posterior tree set by extracting node heights corresponding to the most recent common ancestor of each clade across the posterior sample. The width of each violin represents the density of tMRCA estimates, with wider regions indicating higher posterior probability. Central points indicate the median estimate, and boxes represent the 95% highest posterior density (HPD) intervals. The temporal distribution of tMRCA estimates reveals a gradient of introduction events, with Clade 3 representing the earliest introduction, followed by Clade 2 and subsequently Clade 1. The partial separation and limited overlap among distributions support multiple independent introductions of the BR-2015–2024 lineage into Colombia.
