# Supplementary material for: Phylogeographic dynamics of oropouche virus in the Colombian Amazon: Evolutionary insights in a climatic context
Source: PLoS Negl Trop Dis. 2026 Jul 14;20(7):e0013810. doi: 10.1371/journal.pntd.0013810 (PMC13387556; doi:10.1371/journal.pntd.0013810)
Supplement: S1 Table — Clinical manifestations were compared between patients infected with the PE/CO/EC-2008–2021 lineage and those infected with the BR-2015–2024 lineage using chi-square analysis. No statistically significant differences were observed between lineages. (DOCX) [file pntd.0013810.s005.docx]

**Supplementary work: Phylogeographic Dynamics of Oropouche Virus in the Colombian Amazon: Evolutionary Insights in a Climatic Context.**

Laura S. Perez-Restrepo^1,4^, Jaime Usuga^1,4^, Alejandro Vasquez^1,4^, Lina Yepes^1,4^, Daniel Limonta^2^, Cole Knuese^2^, Isabel Moreno^1,4^, Vanessa Vargas^1,4^, Manuel Gonzalez-Ramirez^1^, Michael G. Berg^3,4^, Mary Rodgers^3,4^, Gavin A. Cloherty^3,4^, Juan P. Hernandez-Ortiz^1,4^*, Jorge E. Osorio^1,2,4^

**S1 Table. Chi-square comparison of clinical manifestations between OROV lineages.**

| **Clinical manifestation** | **χ²** | **p-value** |
| --- | --- | --- |
| Headache | 0.000 | 1.000 |
| Diarrhea | 0.220 | 0.639 |
| Skin rash | 0.080 | 0.778 |
| Muscle pain | 0.000 | 1.000 |
| Bone pain | 0.442 | 0.506 |
| Abdominal pain | 1.363 | 0.243 |
| Chills | 0.000 | 1.000 |

**Table S1. Chi-square comparison of clinical manifestations between OROV lineages.**

**Clinical manifestations were compared between patients infected with the PE/CO/EC-2008–2021 lineage and those infected with the BR-2015–2024 lineage using chi-square analysis. No statistically significant differences were observed between lineages.**
